# Virologic outcomes on dolutegravir-, atazanavir-, or efavirenz-based ART in urban Zimbabwe: A longitudinal study

**DOI:** 10.1371/journal.pone.0293162

**Published:** 2024-02-23

**Authors:** Tinei Shamu, Matthias Egger, Tinashe Mudzviti, Cleophas Chimbetete, Justen Manasa, Nanina Anderegg

**Affiliations:** 1 Newlands Clinic, Harare, Zimbabwe; 2 Institute of Social and Preventive Medicine, University of Bern, Bern, Switzerland; 3 Graduate School of Health Sciences, University of Bern, Bern, Switzerland; 4 Centre for Infectious Disease Epidemiology and Research, School of Public Health, University of Cape Town, Cape Town, South Africa; 5 Population Health Sciences, Bristol Medical School, University of Bristol, Bristol, United Kingdom; 6 Department of Pharmacy and Pharmaceutical Sciences, University of Zimbabwe, Harare, Zimbabwe; 7 Innovation Hub, University of Zimbabwe, Harare, Zimbabwe; NIH: National Institutes of Health, UNITED STATES

## Abstract

There are few data from sub-Saharan Africa on the virological outcomes associated with second-line ART based on protease inhibitors or dolutegravir (DTG). We compared viral load (VL) suppression among people living with HIV (PLWH) on atazanavir (ATV/r)- or DTG-based second-line ART with PLWH on efavirenz (EFV)-based first-line ART. We analyzed data from the electronic medical records system of Newlands Clinic in Harare, Zimbabwe. We included individuals aged ≥12 years when commencing first-line EFV-based ART or switching to second-line DTG- or ATV/r-based ART with ≥24 weeks follow-up after start or switch. We computed suppression rates (HIV VL <50 copies/mL) at weeks 12, 24, 48, 72, and 96 and estimated the probability of VL suppression by treatment regimen, time since start/switch of ART, sex, age, and CD4 cell count (at start/switch) using logistic regression in a Bayesian framework. We included 7013 VL measurements of 1049 PLWH (61% female) initiating first-line ART and 1114 PLWH (58% female) switching to second-line ART. Among those switching, 872 (78.3%) were switched to ATV/r and 242 (21.7%) to DTG. VL suppression was lower in second-line ART than first-line ART, except at week 12, when those on DTG showed higher suppression than those on EFV (aOR 2.10, 95%-credible interval [CrI] 1.48–3.00) and ATV/r-based regimens (aOR 1.87, 95%-CrI 1.32–2.71). For follow-up times exceeding 24 weeks however, first-line participants demonstrated significantly higher VL suppression than second-line, with no evidence for a difference between DTG and ATV/r. Notably, from week 48 onward, VL suppression seemed to stabilize across all regimen groups, with an estimated 89.1% (95% CrI 86.9–90.9%) VL suppression in EFV, 74.5% (95%-CrI 68.0–80.7%) in DTG, and 72.9% (95%-CrI 69.5–76.1%) in ATV/r at week 48, showing little change for longer follow-up times. Virologic monitoring and adherence support remain essential even in the DTG era to prevent second-line treatment failure in settings with limited treatment options.

## Introduction

Antiretroviral therapy (ART) has become more effective, more affordable, and safer since the advent of combination therapy in the mid-90s [1]. Currently, the integrase inhibitor dolutegravir (DTG) in combination with two nucleoside reverse transcriptase inhibitors (NRTIs) is recommended by the World Health Organisation (WHO) for people living with HIV (PLWH) initiating ART and for second-line ART among PLWH who experienced virologic failure on regimens that did not include DTG [2]. Previously, efavirenz (EFV) was recommended for first-line, and the ritonavir-boosted protease inhibitors atazanavir (ATV/r) or lopinavir (LPV/r) for second-line ART, always combined with two NRTIs. DTG achieves earlier virologic suppression compared to ritonavir-boosted protease inhibitors in ART-naïve PLWH, has fewer toxicities and may have a lower or similar incidence of resistance among PLWH receiving second-line ART [2–4]. Compared with efavirenz, first-line DTG had superior viral suppression over 96 weeks, was protective against drug resistance, and led to fewer discontinuations [5–7].

The success of second-line ART depends on several factors. Poor adherence to treatment has been identified in many studies as the critical determinant of virologic failure and the emergence of drug resistance [8, 9]. A high viral load, a low CD4 cell count or advanced HIV infection at the time of switching, concomitant treatment for tuberculosis, and younger age are also associated with poorer outcomes on second-line ART [10–13]. The availability of DTG in a fixed dose combination with lamivudine (3TC) and tenofovir disoproxil fumarate (TDF), commonly referred to as TLD, may improve treatment compliance and lead to better virologic suppression rates among people living with HIV (PLWH) on second-line ART [2].

Compared with boosted protease inhibitors for ART naïve and treatment-experienced PLWH (for whom first-line therapy was failing) with background NRTI resistance, DTG was neither inferior nor superior for viral suppression [14–16]. In PLWH switching to DTG-based second-line ART, viral replication is typically reduced to undetectable levels within the first four to 24 weeks of therapy [17, 18]. However, long-term real-world virologic suppression and clinical outcomes after switching to second-line ART are less well documented. Indeed, there is a lack of data on the outcomes associated with second-line ART based on boosted protease inhibitors and based on DTG-containing regimens, particularly in sub-Saharan Africa. In the context of an urban HIV care and treatment program in Zimbabwe, we examined virologic suppression in adolescents and adults switching to second-line ART due to virologic failure, assessed differences between DTG-based and ATV/r-based second-line regimens, and contrasted second-line suppression rates with those of PLWH starting EFV-based first-line ART.

## Methods

### Setting and data

We analyzed data of individuals receiving ART at Newlands Clinic in Harare, Zimbabwe. Newlands Clinic is an outpatient HIV referral centre with approximately 7,300 individuals in care as of June 2022. The clinic provides comprehensive HIV care to clients of low socio-economic status, including ART, laboratory monitoring, psychosocial support, reproductive health care and other ancillary services. It is supported by the Ruedi Luethy Foundation, a Swiss-based private voluntary organization, and part of the International epidemiology Databases to Evaluate AIDS (IeDEA) in Southern Africa. More details on the clinic’s activities are provided elsewhere [19, 20]. From 2010 to 2019, EFV-based ART was predominantly used as first-line ART and ATV/r as second-line ART at Newlands Clinic. Since 2019, DTG-based ART has been used both in first- and second-line ART [2, 21].

The present analysis was based on the database compiled from the clinic’s electronic medical records system. The system is password protected, and only approved clinic staff can log in and view patient records in accordance with the level of permission. The database contains longitudinal data of individuals receiving ART, including demographic information, CD4 cell counts, and HIV-1 RNA viral loads. Viral loads were typically measured at the time of starting or switching (baseline). After baseline, viral loads were measured at weeks 12 and 24 and then every 24 weeks. Measurements were done using the COBAS Ampliprep/Taqman48 platform and Roche HIV-1 version 1.0 kits (Roche Diagnostics International Ltd, Forrenstrasse 2, Rotkreuz, Zug, 6343, Switzerland). CD4 counts were measured at baseline using a Partec Cyflow Counter II machine with CD4 Easy Count reagents by Sysmex (Sysmex Europe SE, Bornbarch 1, 22848 Norderstedt, Germany). All tests were provided to PLWH receiving care at Newlands Clinic free of charge, similar to the public sector after viral load measurements became widely available in Zimbabwe [22].

At Newlands Clinic, PLWH receiving first-line ART whose HIV viral loads were above 200 copies/mL underwent intensive adherence counselling. Intensive counselling was either offered by the attending nurse or by the mental health department through various models including one-on-one counselling or an enhanced adherence group counselling intervention [23]. The viral load measurements were repeated after three months. If the viral load remained above 200 copies/mL, such individuals were switched to second line ART. The threshold for switching differed from the national standard of care which recommended switching at viral loads above 1000 copies/mL [21]. HIV drug resistance profiles were not recommended prior to switching from first to second line ART.

### Ethics statement

Individuals enrolling into care at Newlands Clinic provided informed written consent that allows the use of data accumulating during their routine care for research under the IeDEA collaboration. IeDEA was approved by the Medical Research Council of Zimbabwe (MRCZ No. A1336) [24]. The study data were stored in a password protected electronic medical records system and were accessed on the 30^th^ of August 2022 by authorised clinic personnel. The abstracted data were supplied to the statistical analysis team as de-identified records that could not be traced to individuals.

### Inclusion criteria

We included PLWH aged 12 years and older who had at least 24 weeks of follow-up after initiating an EFV-based first-line ART between February 2013 and June 2022 or switching from an NNRTI-based first-line to a DTG- or ATV/r-based second-line ART due to virologic failure (viral load above 200 copies/mL).

### Outcome

The outcome of interest was viral load suppression (HIV viral load <50 copies/mL) at week 12, 24, 48, 72 and 96 after baseline, in line with the clinic visits with scheduled VL measurements. We allowed for a one-month window around these time points to account for shifts in visit dates. For example, the “Week 24” measurements included VL measurements done 24 ± 4 weeks after baseline. If several measurements were available, the one closest to the visit date was chosen for analysis. Some VL measurements fell between two windows (for example, a measurement drawn 35 weeks after baseline was neither assigned to “Week 24” nor to “Week 48”). These measurements were used to impute missing VL information when a patient did not have a VL measurement for a visit date but did have a measurement right before and after the window around the visit date. We assumed that the missing measurement indicated suppressed or unsuppressed VL if both measurements (before and after) showed a suppressed or unsuppressed VL. If the measurements before and after were different (suppressed and unsuppressed), we did not impute the VL suppression information for the visit date. As this imputation approach does not allow the imputation of viral blips, we also examined results based on complete cases only.

### Explanatory variables

Explanatory variables included the time of the VL measurement (as defined above as Week 12, Week 24, Week 48, Week 72, or Week 96 after baseline), the ART regimen, the patient’s sex, age and CD4 cell count at baseline. Age at baseline was grouped into 12–19 years, 20–29, 30–39 years and ≥40 years. CD4 cell count at baseline was grouped into 0–199 cells/mm^3^, 200–349 cells/mm^3^ and ≥350 cells/mm^3^. ART regimens included “EFV” for first-line ART, and “ATV/r” and “DTG” for second-line ART. The minimal supporting data is available in S1 Data.

### Statistical analysis

We calculated crude proportions of viral load suppression after baseline by ART regimen. We then estimated the probability of viral load suppression with multivariable logistic regression models. We used a Bayesian framework with weakly informative priors to enhance inference in situation with low numbers when fitting interactions (e.g., few VL measurements in high baseline CD4 categories for second-line regimens). To account for the dependence of viral load measurements within individuals, we included a random intercept by individual. As fixed effects, we included the covariates ART regimen, time of the VL measurement, sex, age and CD4 cell count. We checked if the inclusion of any two-way interaction between fixed-effect covariates improved the model fit in two steps. First, we compared model fits of the simple model without any interactions to alternative models containing one single two-way interaction. All two-way interactions for whom the comparison to the simple model showed a difference in expected log pointwise density that was larger two times its standard error were considered "eligible” for inclusion in the final model [25]. In the second step, we looked at all possible combinations of the eligible two-way interactions. The final model was the one with the best fit (again, according to the expected log-density). For more information about the statistical models and prior distributions, see S1 Text.

We report adjusted odds ratios (aOR) for VL suppression and crude and predicted proportions of VL suppression with 95% credible intervals (CrIs). For both aORs and predicted probabilities, we report marginalized (“sample-averaged”) estimates derived by integrating over the distribution of random effects. Next to reporting predicted proportions stratified by all levels of covariates, we also report them standardized and non-standardized by time and treatment regimen. For standardized predictions, we predicted the probability of VL suppression at weeks 12, 24, 48, 72, and 96 for every study patient, assuming they were on DTG, then assuming they were on ATV/r and finally on EFV. We then averaged all the predicted probabilities of viral load suppression for each treatment regimen and time point. Non-standardized predictions consisted of predicting the probability of VL suppression for the observed data only.

## Results

Overall, we included 2163 PLWH (59.9% female), of whom 1114 were switching to second-line ART, and 1049 were initiating first-line ART (Table 1). PLWH switching to second-line ART were younger (median age 29 years; IQR 19–42) than those starting first-line ART (36 years; IQR 28–44). Among the second-line ART individuals, 872 (78.3%) switched to ATV/r-based ART, while 242 (21.7%) switched to DTG-based ART (Table 1). PLWH switching to ATV/r-based second-line ART had lower CD4 cell counts at switch than those switching to DTG or starting an EFV-based first-line regimen (Table 1). The 2163 PLWH contributed 7413 VL measurements, of which (7181, 96.9%) were observed and 232 (3.0%) imputed. Around three-quarters of individuals had a VL measurement at shorter follow-up times (weeks 12, 24, 48), while this proportion was lower for longer follow-up times (Table 1). For DTG, fewer individuals had a VL measurement at longer follow-up times than the other two regimens. As indicated by the later calendar years of starting/switching ART for DTG, this was mainly due to the follow-up time of people switching to DTG being too short to contribute VL measurements for later weeks (Table 1). Only a few individuals were lost to follow-up or died during the follow-up time relevant to this study (Table 1).

**Table 1:** Baseline demographic and clinical characteristics of participants by treatment regimen.

	First line	Second line		Overall
	EFV	ATV/r	DTG	
**Total**	1049	872	242	2163
**Female**	645 (61.5%)	506 (58.0%)	145 (59.9%)	1296 (59.9%)
**Male**	404 (38.5%)	366 (42.0%)	97 (40.1%)	867 (40.1%)
**Age [years]**				
** Median (IQR)**	36 (28–44)	29 (19–41.25)	28.5 (19–42)	34 (22–43)
** 12–19**	97 (9.2%)	224 (25.7%)	67 (27.7%)	388 (17.9%)
** 20–29**	202 (19.3%)	219 (25.1%)	58 (24.0%)	479 (22.1%)
** 30–39**	348 (33.2%)	161 (18.5%)	49 (20.2%)	558 (25.8%)
** 40+**	402 (38.3%)	268 (30.7%)	68 (28.1%)	738 (34.1%)
**CD4 cell count [cells/mm** ^**3**^ **]**				
** Median (IQR)**	251 (122–363)	182 (73–331)	253 (78–469)	224 (97–360)
** <200**	426 (40.6%)	461 (52.9%)	98 (40.5%)	985 (45.5%)
** 200–349**	331 (31.6%)	219 (25.1%)	51 (21.1%)	601 (27.8%)
** > = 350**	292 (27.8%)	192 (22.0%)	93 (38.4%)	577 (26.7%)
**Previous regimen (for 2**^**nd**^ **line participants)**				
** EFV-based**		631 (72.4%)	205 (84.7%)	
** NVP-based**		241 (27.6%)	29 (12.0%)	
** ATV/r-based**		-	1 (0.4%)	
** Missing**		0	7 (2.9%)	
**Total number with VL measurement**				
** at week 12**	800 (76.3%)	683 (78.3%)	202 (83.5%)	1685 (77.9%)
** at week 24**	884 (84.3%)	722 (82.8%)	175 (72.3%)	1781 (82.3%)
** at week 48**	812 (77.4%)	663 (76.0%)	155 (64.0%)	1630 (75.4%)
** at week 72**	424 (40.4%)	506 (58.0%)	108 (44.6%)	1038 (48.0%)
** at week 96**	627 (59.8%)	565 (64.8%)	87 (36.0%)	1279 (59.1%)
**Year of ART start/switch**				
** Median**	2016	2017	2021	2017
** (IQR)**	(2015–2017)	(2016–2018)	(2020–2021)	(2016–2018)
**≤ 2016**	716 (68.3%)	480 (55.0%)	0 (0%)	1196 (55.3%)
** 2017–2019**	332 (31.6%)	378 (43.3%)	70 (38.9%)	780 (36.1%)
**≥ 2020**	1 (0.1%)	14 (1.6%)	172 (71.1%)	187 (8.6%)
Status at end of follow-up*				
** In care**	988 (94.2%)	832 (95.4%)	230 (95.0%)	2050 (94.8%)
** LTFU**	42 (4.0%)	18 (2.1%)	4 (1.7%)	64 (3.0%)
** Dead**	19 (1.8%)	22 (2.5%)	8 (3.3%)	49 (2.3%)

* LTFU and Dead correspond to individuals who were lost to follow-up or died within the follow-up time relevant to this study. In care corresponds to those who remained in care by week 100 after starting/switching ART or at database closure, whichever came first. EFV, efavirenz; ATV/r, ritonavir-boosted atazanavir; DTG, dolutegravir; IQR, interquartile range; LTFU, lost to follow-up.

**Fig 1:**
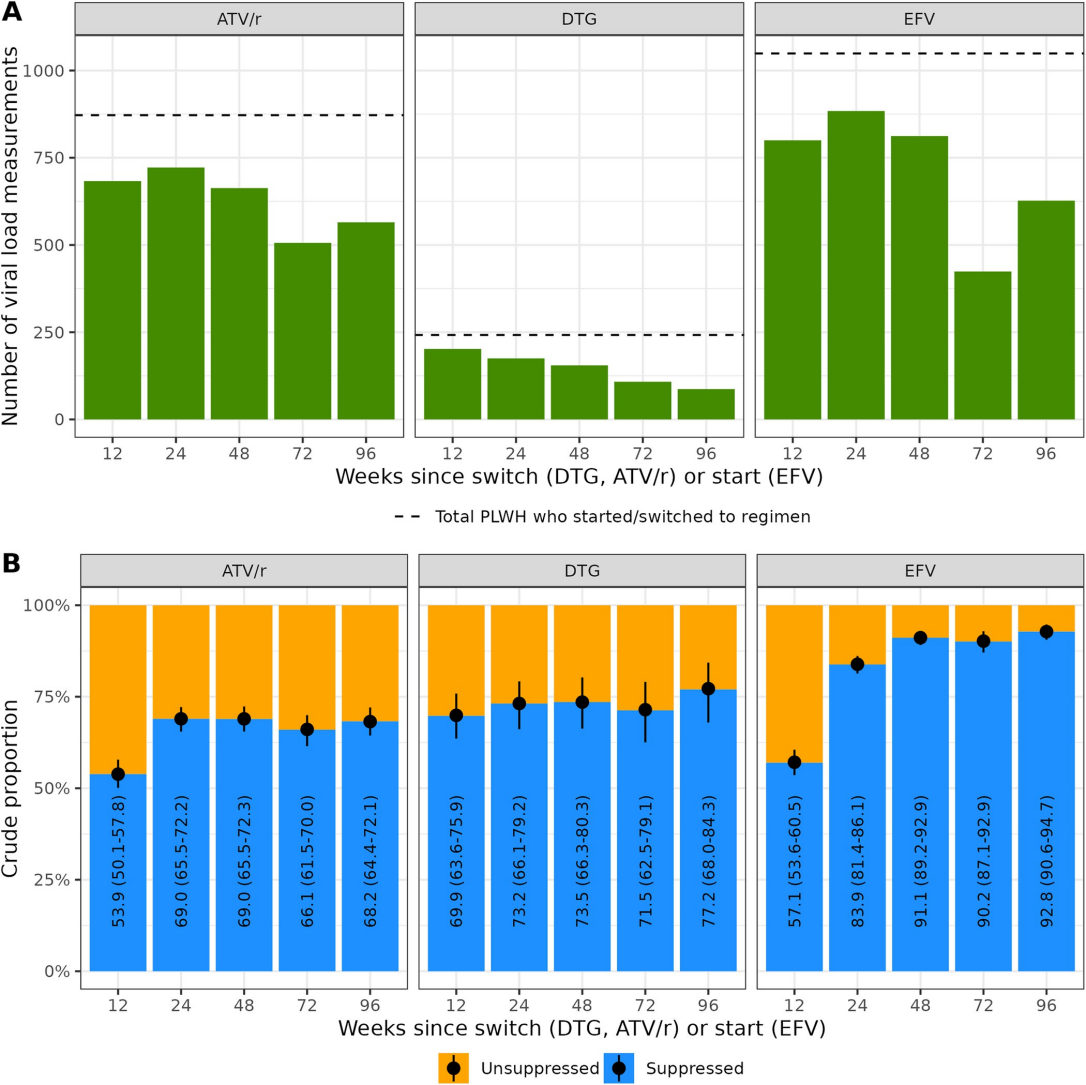
Number of PLWH with viral load measurements (panel A) and crude proportions (with 95% credible intervals) of viral load suppression (panel B) by treatment regimen and time post switch (ATV/r, DTG) or start (EFV) of ART. EFV, efavirenz; ATV/r, ritonavir-boosted atazanavir; DTG, dolutegravir; PLWH = people living with HIV.

Crude rates of virologic suppression were lower on second-line ART than first-line ART (Fig 1). The exception was week 12 when the DTG-based regimen showed higher suppression (crude proportion 69.9%, 95%-CrI 63.6–75.9%) than ATV/r (crude proportion 53.9%, 95%-CrI 50.1–57.8%) and EFV-based regimens (crude proportion 57.1%, 95%-CrI 53.6–60.5%). Among the second-line ART regimens, crude rates of virologic suppression for follow-up times greater than 24 weeks were slightly higher for DTG (73.5%, 95%-CrI 69.7–77.5%) compared to ATV/r (68.2%, 95%-CrI 66.2–70.0%).

Only the two-way interactions between time and ART regimen and between time and age met the criteria for potential inclusion in the final model. The model including only the time-ART regimen interaction, and the model including both the time-ART regimen and the time-age interaction led to similar model fits, with a slightly better fit for the simpler model (S1 Text). The final logistic regression model thus only included the two-way interaction between time and ART regimen. The final model fitted the data well, with a few exceptions when there was large uncertainty due to small numbers (S1 Fig).

**Fig 2:**
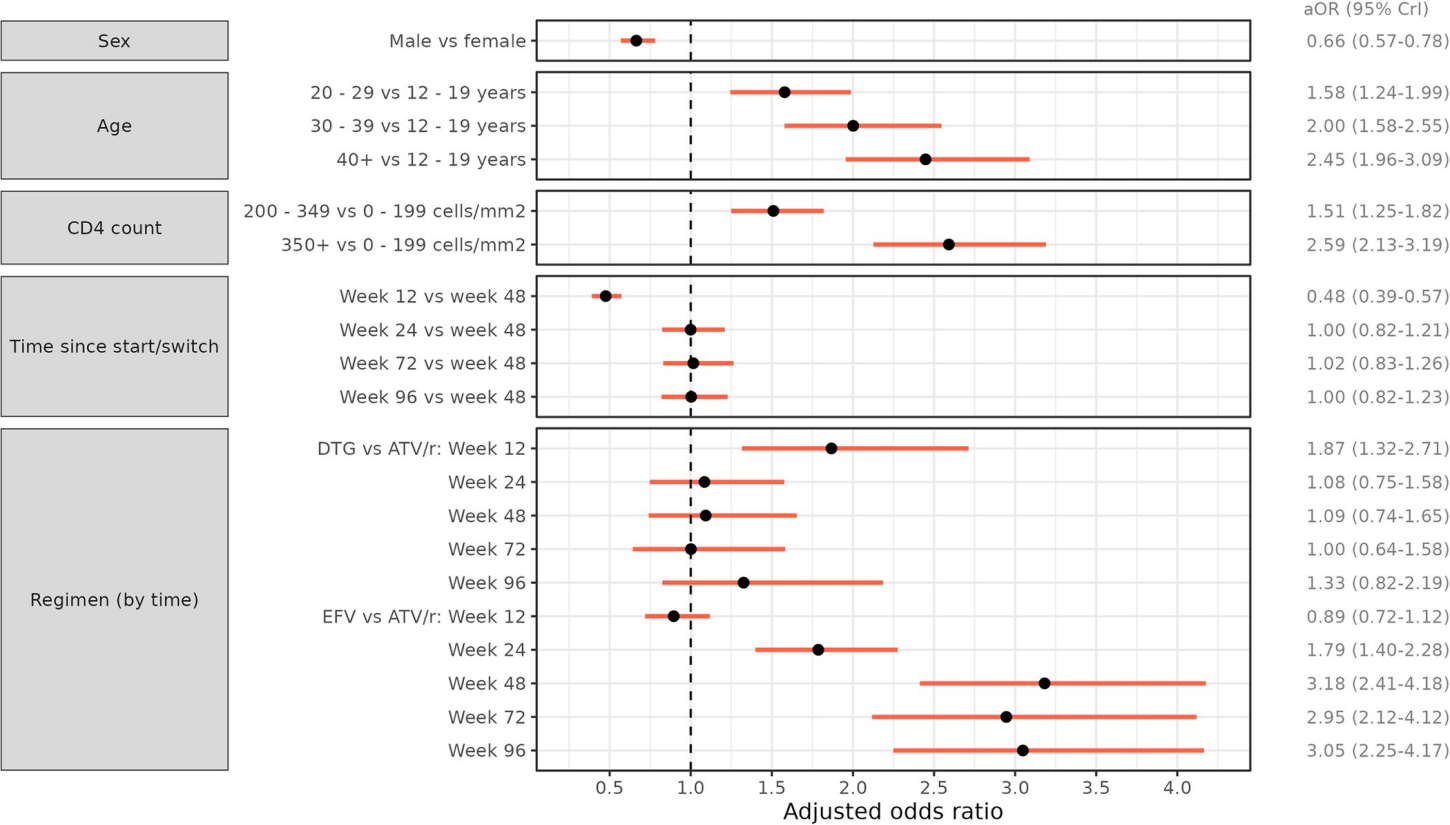
Adjusted odds ratios (aOR) for viral load suppression with 95% (red) credible intervals. Results derived from a Bayesian logistic regression model including a random intercept by patient and the covariates sex, baseline age, baseline CD4 cell count, time since start/switch, and ART regimen. The model also includes a two-way interaction between ART regimen and time since start/switch, so that the aORs comparing ART regimens are shown stratified by time. Estimates are marginalized by integration over the distribution of random effects. EFV, efavirenz; ATV/r, ritonavir-boosted atazanavir; DTG, dolutegravir.

**Fig 3:**
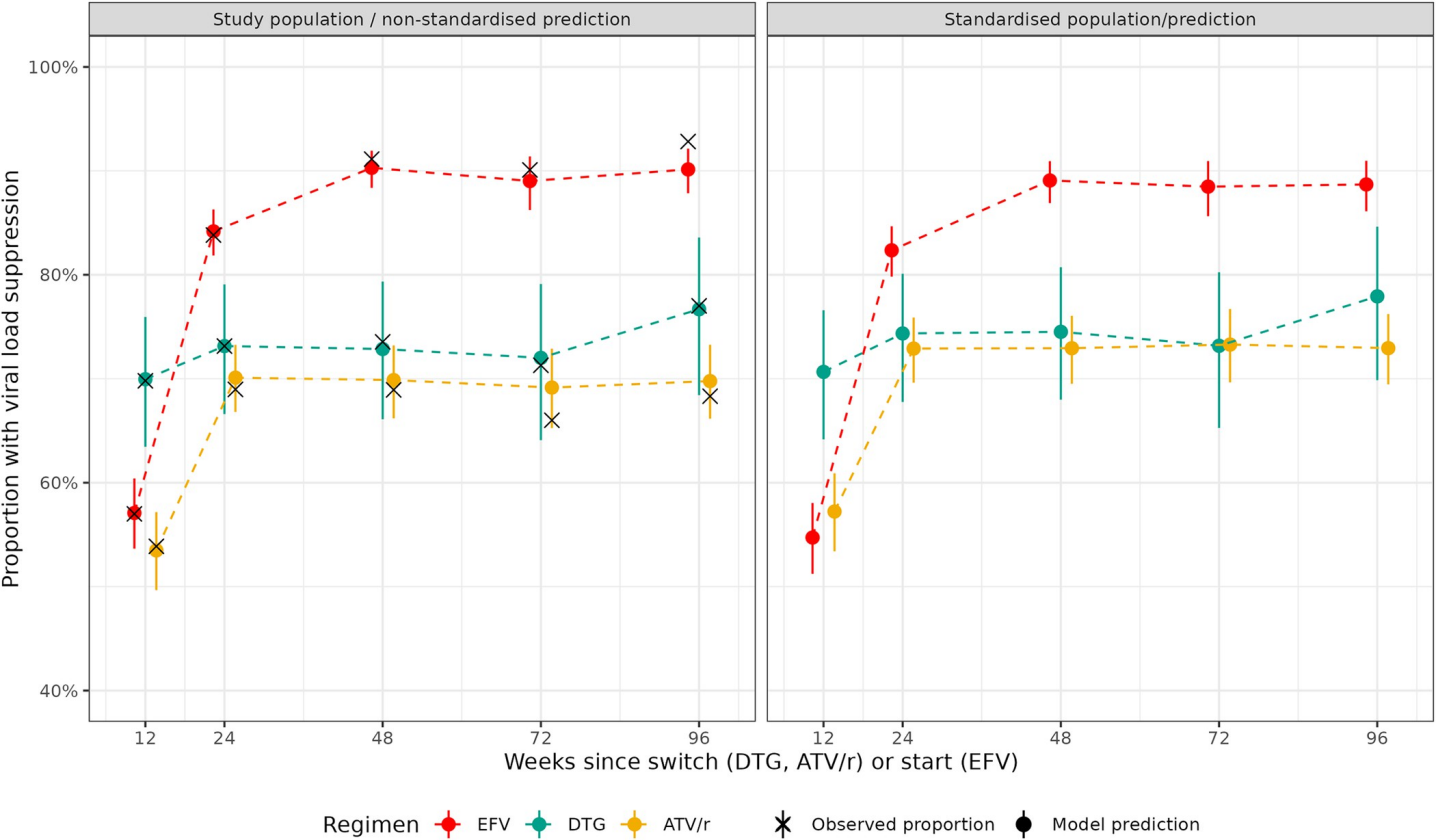
Non-standardized (left panel) and standardized (right panel) predicted proportions and 95% credible intervals of viral load suppression by treatment regimen and time since switch (DTG, ATV/r) or start (EFV). Results from a Bayesian logistic regression model including a random intercept by patient and the covariates sex, baseline age, baseline CD4 cell count, time since start/switch, ART regimen, and a two-way interaction between time since start/switch and ART regimen. Estimates are marginalized by integrating over the distribution of random effects.

The regression model confirmed the higher odds of VL suppression for DTG at short follow-up (aOR 1.87, 95% CrI 1.32–2.71 comparing DTG to ATV/r at Week 12 and aOR 2.10, 95% CrI 1.48–3.00 comparing DTG to EFV, Fig 2). While for DTG, the odds of VL suppression did not increase substantially for longer follow-up periods, they did so for PLWH on ATV/r and even more for those on EFV (Figs 2 and 3). After 24 weeks, the estimated odds of VL suppression were relatively stable for both second-line regimens, and there was no evidence of a difference between DTG and ATV/r. For EFV, the odds of VL suppression also reached a stable plateau but slightly later, at 48 weeks. At that time, the odds of VL suppression for EFV were estimated to be around 3 times higher than those for second-line regimens (Figs 2 and 3). Apart from the differences between first- and second-line regimens, the odds of VL suppression were higher for females and increased with age and CD4 cell count (Fig 2). VL suppression was lowest for adolescents, with estimated suppression rates mostly below 75% in adolescents switching to second-line ART and below 60% for those switching with CD4 cell counts below 200 cells/mm^3^ (S1 Fig). Results from sensitivity analysis including complete cases only were almost identical to the results from the main analysis (S2 Fig).

Standardized and non-standardized differences in VL suppression rates between regimens were overall similar, but standardization attenuated differences between the two second-line regimens (Fig 3). At week 48, standardized predicted proportions of VL suppression were 89.1% (95% CrI 86.9–90.9%) for EFV, 74.5% (95%-CrI 68.0–80.7%) for DTG, and 72.9% (95%-CrI 69.5–76.1%) for ATV/r (Fig 3). Corresponding non-standardized proportions of VL suppression at week 48 were 90.3% (95%-CrI 88.4–91.9%) for EFV, 72.9% (95%-CrI 66.1–79.3%) for DTG, and 69.9% (95%-CrI 66.2–73.2%) for ATV/r (Fig 3).

## Discussion

In this longitudinal analysis of routine clinical data collected in an urban ART program in Zimbabwe, we examined virologic suppression among PLWH switching to DTG- or ATV/r-based second-line ART after experiencing virologic failure, and PLWH starting ART on EFV-based first-line regimens. Suppression rates were highest for first-line PLWH receiving EFV-based ART from weeks 24 through 96. Notably, at week 12, DTG-based ART exhibited superior suppression compared to EFV-based ART, affirming its faster efficacy in viral replication control, even when used in second-line ART [3, 4]. Older age and higher CD4 cell count at switch or start were associated with higher odds of virologic suppression. In contrast, male sex was associated with reduced odds of virologic suppression across all regimens.

Our study took advantage of a long-standing, well-characterized cohort with high retention in care and reliable data collection [20]. This is reflected in the fact that we had little loss to follow-up over the study period and no missing CD4 counts, which is rare for observational cohorts in similar settings. Another strength lies in using a Bayesian approach, which allowed us to test for many interactions and calculate standardized probabilities of viral load suppression that allow more informative comparisons between treatment regimens correcting for differences in characteristics between first- and second-line individuals.

Our study’s limitations include the absence of objective measures of treatment adherence and baseline drug resistance profiles. Also, not all individuals had VL measurements taken at all time points, especially in later weeks and individuals switching to DTG. As loss-to-follow-up was rare, this was attributed mainly to two factors. Firstly, a shift in the visit schedule was a common cause. Measurements were limited to within a 1-month window of the designated week of interest (e.g., “week 24” included VL measurements done during weeks 24±4). Despite the potential for larger windows including more measurements, we opted for this constraint to avoid distortion of suppression rates. With this however, measurements from individuals whose visit schedule was shifted by more than 4 weeks were excluded from the analysis (although these measurements were used for imputing missing measurements in few cases). We opted against using methods like multiple imputation after confirming with Newlands Clinic personnel that they were confident there was no systematic difference between individuals with a shift in the visit schedule and those without. The second reason for the absence of a VL measurement at a specific week was insufficient follow-up time. This was particularly relevant to individuals who switched to DTG, introduced on large-scale only in 2019. Unlike EFV and ATV/r, which were mainly used before 2019, allowing most participants to have ≥2 years of follow-up, many DTG switchers could only contribute VL measurements to the earlier weeks. This could also happen with the other two regimens, for example, if someone initially on EFV was programmatically switched to DTG after a few weeks. The distinct periods during which the different regimens were used are another limitation. Many individuals switched to DTG-based second-line during the Covid-19 national lockdown period, and we cannot exclude residual confounding by the Covid-19 pandemic or other factors that might have changed over time.

Many of the PLWH on second-line ART will probably have a history of poor adherence, which led to virologic failure on first-line ART and, in some individuals, could have led to the development of drug resistance. However, NNRTI pretreatment resistance likely played a more significant role in the last decade [26–29]. Second-line virologic failure (DTG or ATV/r) may more likely be related to poor treatment compliance based on literature showing almost no pretreatment or transmitted drug resistance among protease inhibitor- and INSTI-naïve PLWH in Zimbabwe and the demonstrated efficacy of both DTG and protease inhibitors with suboptimal NRTI backbones [15, 27, 30–32]. In addition, a longer duration of ART is associated with a higher likelihood of treatment failure [33, 34]. Even though both DTG and ATV/r are potent antiretrovirals, with DTG being superior to EFV for both viral suppression and tolerability [5, 7, 35], the overall suppression rates among the second-line regimens were lower than those observed for first-line ART in all age groups, sex, and baseline CD4 cell count strata. This suggests that for PLWH with a history of treatment failure, switching to DTG or ATV/r alone without other adherence support interventions may not be sufficient to reach the programmatic targets of 95% or higher VL suppression [23, 36]. Failure to achieve virologic suppression among PLWH receiving second-line DTG-based ART can expose these individuals to the emergence of drug resistance against DTG and the beginning of transmitted drug resistance to the INSTI class. Several cases of acquired and pretreatment DTG resistance have already been reported [37–39].

Almost half of the participants included in this study had advanced HIV disease with baseline CD4 cell counts <200cells/mm^3^. Although lower CD4 cell counts are the consequence of HIV replication, it is unclear why lower baseline CD4 counts are associated with failure to suppress VL across all the regimens in this study. Higher viral loads at ART commencement are associated with prolonged durations before viral suppression leading to increased odds of virologic failure on NNRTI containing ART [40, 41]. Further, severe immune suppression might indicate a lower commitment to starting/switching ART or engaging in adherence support initiatives, which in turn is likely associated with reduced treatment adherence [23]. Low CD4 cell counts when switching to second-line ART may also result from previous loss to follow-up, treatment interruption, or inconsistent clinic visits. Some studies have also shown a relationship between low baseline CD4 cell counts and subsequent virologic failure, while another study from Mozambique showed an association between higher baseline CD4 cell counts and virologic failure [26, 42, 43].

PLWH switching to second-line ART were younger than those starting first-line. Likewise, younger people were also less likely to achieve virologic suppression across all regimens. Younger age has previously been associated with poorer adherence and virologic failure without accompanying drug resistance [26, 44]. This observation warrants investment in custom treatment support interventions that foster improved treatment adherence and more frequent viral load monitoring among young PLWH with history of treatment failure.

## Conclusion

PLWH receiving second-line ART were less likely to achieve virologic suppression than those on first-line ART, despite switching to robust or even superior regimens. The probability of virologic suppression increased with increasing age and baseline CD4 cell count. Further efforts are needed to enhance treatment adherence among young PLWH and, more broadly, those switching after virologic failure in settings with limited treatment options.

## Supporting information

S1 Data Supporting data available at https://rb.gy/30fslu. (ZIP)

S1 Text Information about Bayesian model fits. (PDF)

S1 Fig Predicted (colour) and crude/observed (black) proportions (with 95% credible intervals) of viral load suppression stratified by all covariates included in the model fit.
(TIF)

S2 Fig Adjusted odds ratios (aOR) for viral load suppression with 95% credible intervals from the main analysis (red) and the sensitivity analysis based on complete cases only (purple). The sensitivity analysis excluded 226 VL measurements which were imputed in the main analysis.
(TIF)
