# Supplementary material for: Virologic outcomes on dolutegravir-, atazanavir-, or efavirenz-based ART in urban Zimbabwe: A longitudinal study
Source: PLoS One. 2024 Feb 23;19(2):e0293162. doi: 10.1371/journal.pone.0293162 (PMC10890724; doi:10.1371/journal.pone.0293162)
Supplement: S1 Text — (PDF) [file pone.0293162.s002.pdf]

S1 Text. Information about Bayesian models fitted.

For variable selection, we first compared the following baseline ("no interaction") model

```
stan_glmer(Suppressed  ~ sex + age + regimen + CD4_count + Week +
                        (1|individual_ID),
                        family = "binomial",
                        prior = normal(0,2.5),
                        prior_intercept = normal(0,2.5),
                        prior_covariance = decov(regularization = 1, concentration = 1, shape =1, scale = 1)
```

to alternative models. The alternative models had the same model-structure as the baseline model above (i.e., included all fix-effects covariates week, regimen, sex, age, cd4 count - and a random intercept per individual), but included additionally a single two-way interaction between any of the fixed-effects covariates. All alternative models were individually compared against the baseline model containing no interactions (see Step 1 in table below). For the comparison in Step 1, positive differences in ELPD indicate that the model including the specific two-way interaction fitted better than the simple model, negative differences indicate that the simple model fits better. Only two-way interactions leading to a positive difference in ELPD > 2* standard error were considered "eligible" for step 2. This applied to two different alternative models, one including a interaction between age and week, and one including an interaction between regimen and week. In Step 2, models with all possible combinations of eligible two-way interactions are compared to each other. In step 2, the best model will have difference in ELPD of 0 and the other differences correspond to the comparison of the specific model to the best model. The best model (with ELPD = 0) is than used as final model.

**Step 1**	**Difference in ELPD**	**Standard error**
Regimen:Week vs. no interaction	+62,0694	18,55541
Age:Week vs. no interaction	+38,7228	15,00842
Sex:Age vs. vs. no interaction	+6,66894	13,01469
CD4 count:Week vs. vs. no interaction	+4,49399	14,00591
Sex:Week vs. no interaction	+1,80612	13,90913
Regimen:Age vs. no interaction	+0,19347	13,69658
Regimen:CD4 count vs. no interaction	-0,04289	13,23341
Sex:CD4 count vs. no interaction	-1,24851	12,63944
Age:CD4 count vs. no interaction	-3,63069	13,86179
Regimen:Sex vs. no interaction	-14,928	13,63342
**Step 2**		
Regimen:Week vs. Regimen:Week	0	0
Regimen:Week + Age:Week vs. Regimen:Week	-10,8092	14,68336
Age:Week vs. Regimen:Week	-23,3466	18,03875
No interaction vs. Regimen:Week	-62,0694	18,55541

The final model used for the analysis based on the model selection above was the model including an interaction between regimen and week:

```
stan_glmer(Suppressed  ~ sex + age + regimen + CD4_count + Week + regimen:Week,
           (1|individual_ID),
           family = "binomial",
           prior = normal(0,2.5),
           prior_intercept = normal(0,2.5),
           prior_covariance = decov(regularization = 1, concentration = 1, shape =1, scale = 1)
```
