# Supplementary figures and images for: Virologic outcomes on dolutegravir-, atazanavir-, or efavirenz-based ART in urban Zimbabwe: A longitudinal study
Source: PLoS One. 2024 Feb 23;19(2):e0293162. doi: 10.1371/journal.pone.0293162 (PMC10890724; doi:10.1371/journal.pone.0293162)

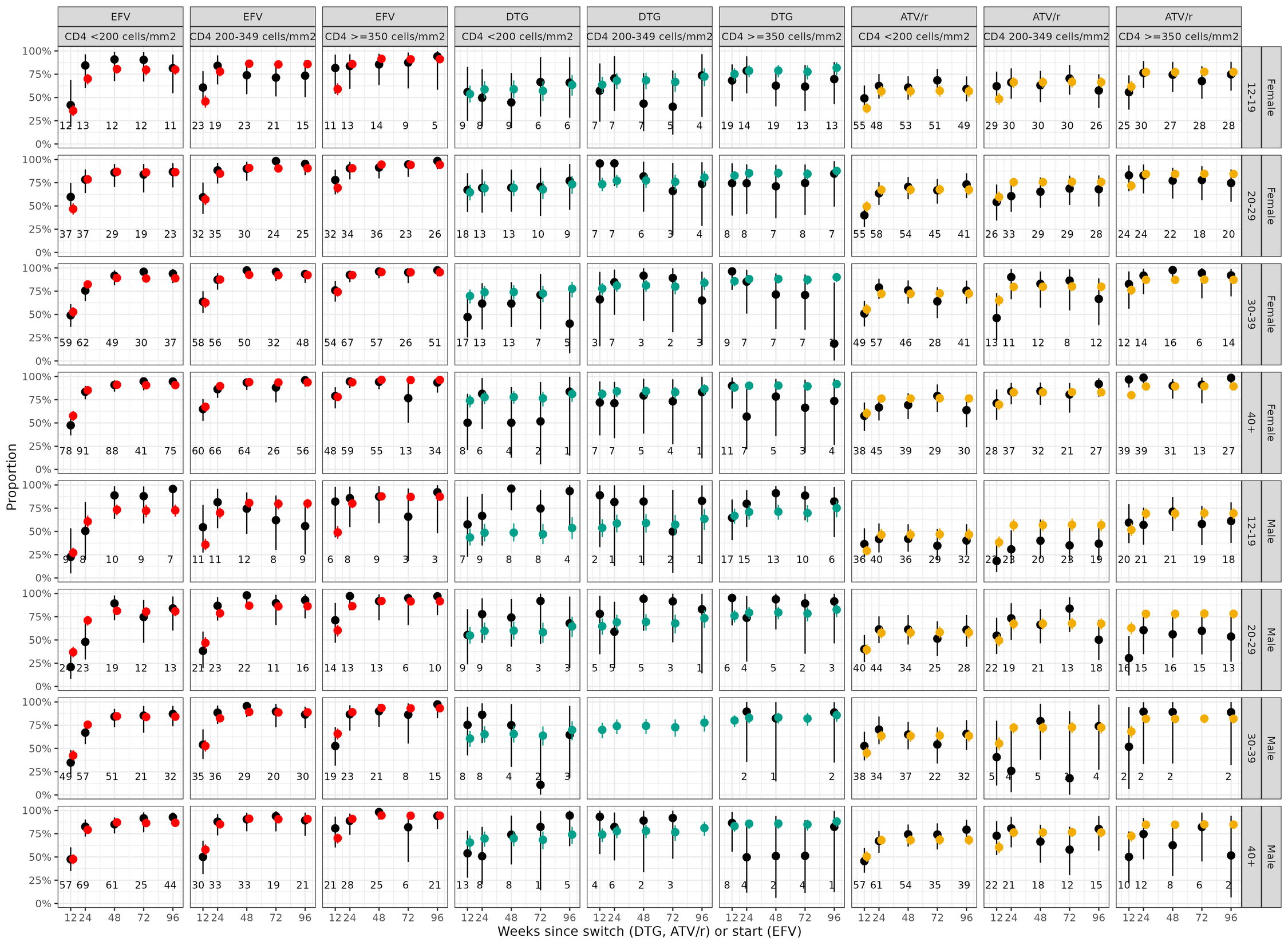

Supplement: S1 Fig — Predicted (colour) and crude/observed (black) proportions (with 95% credible intervals) of viral load suppression stratified by all covariates included in the model fit. (TIF) [file pone.0293162.s003.tif]

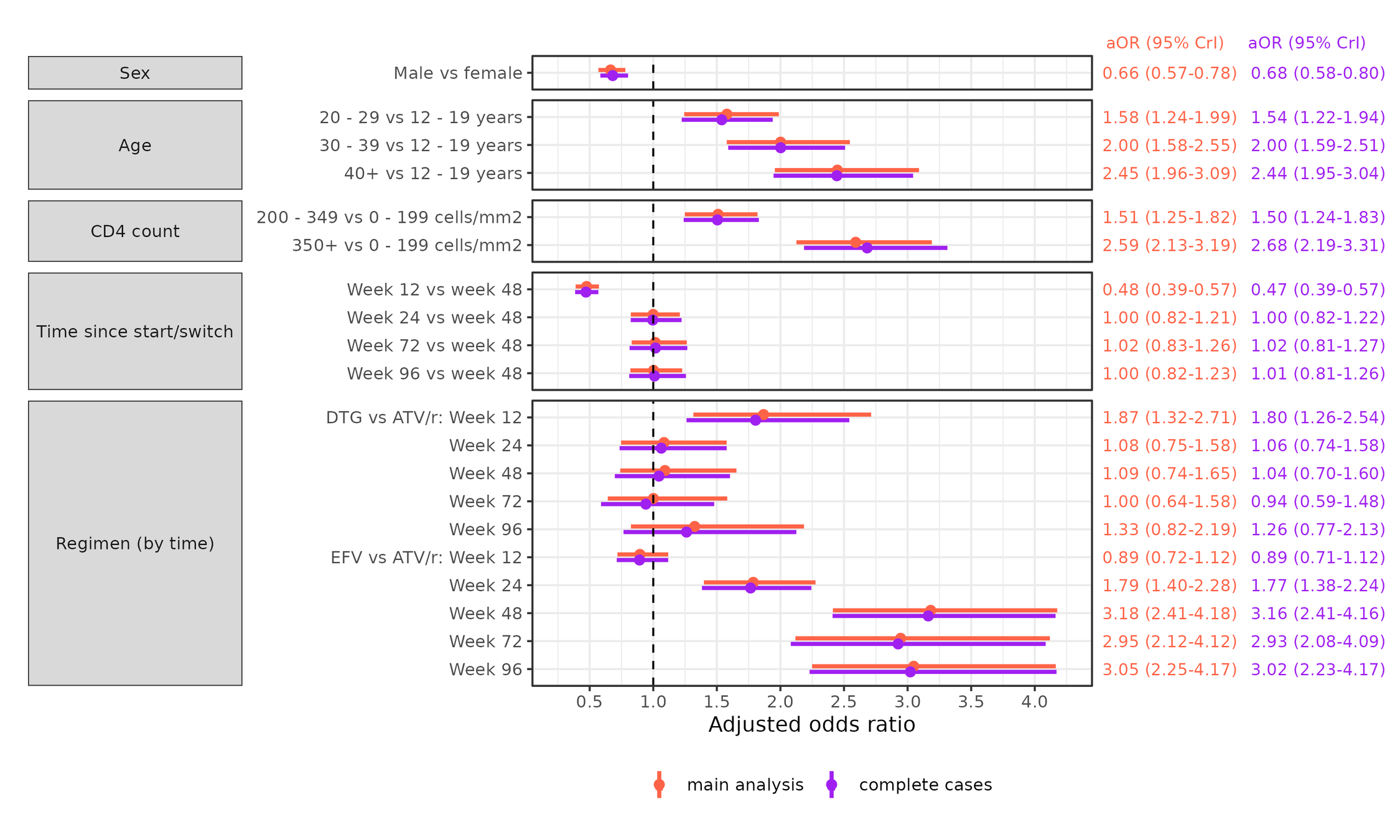

Supplement: S2 Fig — The sensitivity analysis excluded 226 VL measurements which were imputed in the main analysis. (TIF) [file pone.0293162.s004.tif]
